# Seed germination in *Narcissus yepesii* (Amaryllidaceae): clinal variation in the morphophysiological dormancy levels

**DOI:** 10.1093/aobpla/plaa060

**Published:** 2020-11-17

**Authors:** Elena Copete, Miguel A Copete, Pablo Ferrandis, José M Herranz

**Affiliations:** 1 E.T.S.I.A.M. Department of Plant Production and Agricultural Technology, University of Castilla-La Mancha, Albacete, Spain; 2 Institute of Botany, University of Castilla-La Mancha, Albacete, Spain

**Keywords:** Gibberellic acid, *Narcissus alcaracensis*, *Narcissus longispathus*, radicle emergence, secondary dormancy, seed storage, seed stratification

## Abstract

Seed dormancy classes determine both population and species-level processes which can be crucial in the life cycle of many plants. However, there are no studies of a dormancy cline between levels of morphophysiological dormancy (MPD). We aimed to determine the class of seed dormancy of *Narcissus yepesii* exhibits in order to explore links between different dormancy levels, previously characterized in two closely related phylogenetic congeners, *N. alcaracensis* and *N. longispathus*. Experiments were carried out under both near-natural temperature and controlled laboratory conditions. The parameters calculated were mean embryo length, radicle and shoot emergence percentages. The effects of different periods of storage; and different periods with or without GA_3_ of warm, cold or warm plus cold were analysed. The *Narcissus* populations from the Baetic System of mountain ranges in south-eastern Spain show clinal variation in a northeast–southwest gradient from intermediate to non-deep complex MPD, through the coexistence of intermediate and non-deep complex MPD in *N. yepesii* (21 % and 74 %, respectively). In addition, 54 % of stored seeds were able to show both levels of MPD. *Narcissus yepesii* occupies an intermediate position between *N. alcaracensis* and *N. longispathus* in the geographical distribution and in the clinal germination ranges. It strongly suggests an evolutionary gradient, which connects the intermediate complex MPD with the non-deep complex MPD in southern Iberian daffodils. This is the first study showing a gradient in the evolution between levels of MPD. Our results demonstrate a cline in these levels in response to both an environmental gradient and genetic differences.

## Teaser

This is the first study showing a gradient in the evolution between levels of morphophysiological dormancy (MPD) in response to both an environmental gradient and genetic differences. This dormancy class occurs when seeds with underdeveloped embryos have physiological components preventing germination as well. Previously, two different levels of MPD: intermediate and non-deep complex were characterized in two closely related phylogenetic congeners, *N. alcaracensis* and *N. longispathus*, respectively. Our results demonstrate a clinal variation through the coexistence of intermediate and non-deep complex MPD in *N. yepesii* (21 % and 74 %, respectively), which occupies a middle geographical position between the other two taxa.

## Introduction

Seed germination and the recruitment of new individuals from seeds are currently recognized as crucial phases of the plant’s life that are strongly dependent on environmental filters which act as signals breaking seed dormancy (Donohue *et al.* 2010). As a result, seed germination in nature is often restricted to particular locations, which provide appropriate environmental requirements, to the point that different environmental signals for seed germination can reflect habitat adaptations (Grubb 1977; Vandelook and Van Assche 2008). The dormancy class can influence both population and species-level processes such as colonization, adaptation, speciation and extinction (Willis *et al.* 2014). Between the different dormancy classes, morphophysiological dormancy (MPD) is the most difficult to overcome because embryos must attain a critical length before germination can take place and growth of underdeveloped embryos requires specific durations of warm and/or cold stratification depending on the level of MPD (Baskin and Baskin 2014).

In the short term, the successful of germination requires specific dormancy-breaking cues that have to be present in the habitat. However, trait evolution from one to other level of MPD can occur as adaptation to environment in the long term. The evolution of levels of MPD can be consequence of divergence of the taxa in disjunct congeners, such as North American–Asian species of *Osmorhiza* with non-deep and deep complex, respectively (Walck *et al.* 2002) and Eurasian–North American species of *Sambucus* with intermediate complex and deep simple, respectively (Hidayati *et al.* 2000b). Also, this evolution has been studied in close-related species, whose populations are separated by just a few kilometres, such as *Narcissus alcaracensis* with intermediate complex MPD (Herranz *et al.* 2013b) and *N. longispathus* with non-deep complex MPD (Herranz *et al.* 2013a).

*Narcissus* (Amaryllidaceae) is a genus of perennial geophytes whose main centre of diversity is the Iberian Peninsula and Morocco, but it also occurs elsewhere along the coasts of the Mediterranean and the European Atlantic. Taxonomic difficulties arise from weak reproductive barriers, resulting in the widespread occurrence of hybridization and a high morphological variability. Consequently, the number of wild daffodil species varies considerably between different taxonomic treatments from 36 differentiated by Zonneveld (2008) to 64 recognized by Blanchard (1990).

**Figure 1.**
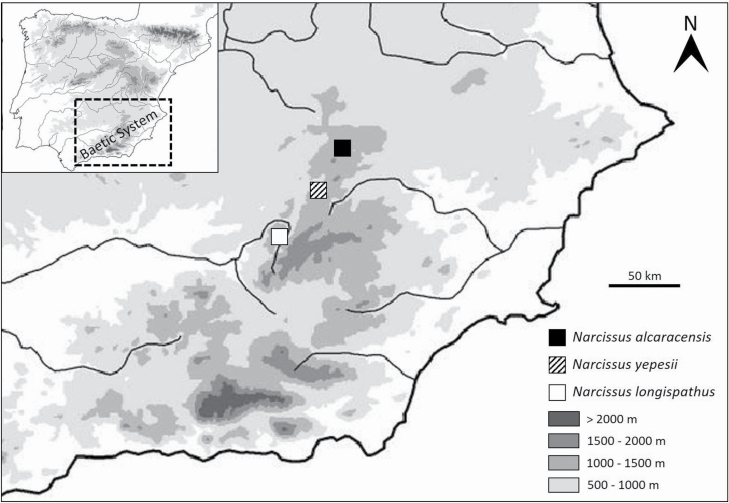
Seed populations in Baetic System: *N. alcaracensis* in Alcaraz Mountains (Albacete province); *N. yepesii* in Segura Mountains (Jaén province); and *N. longispathus* in mountains of Cazorla, Castril and Magina (Jaén province).

A new taxon belonging to this group has been described in the last decades: *Narcissus yepesii* from the *Pseudonarcissi* section (Ríos-Ruiz *et al.* 1999). This daffodil grows along the banks of small permanent streams in small populations, and the number of individuals ranges between several dozens to a few hundred. Its main distribution area occurs in Segura Mountains, which are part of the Baetic System of mountain ranges (in the south-east of the Iberian Peninsula). The other two species above mentioned from the same section are found in nearby areas from the same Baetic mountain system: (i) *N. alcaracensis* in the Alcaraz Mountains (Juan-Vicedo *et al.* 2018), and (ii) *N. longispathus* in mountains of Cazorla, Castril and Magina (Hernández-Bermejo *et al.* 2006). The Euclidean distance between the location of the populations of *N. alcaracensis* and *N. longispathus* is ~97 km, while *N. yepesii* grows in the middle (~37 km from *N. alcaracensis* and 60 km from *N. longispathus*). A clinal geographic variation in a northeast–southwest gradient exists between these three species (Fig. 1) and this geographical variation involves a smooth increase of mean annual temperature (from 13.1 to 15.7 °C), which could influence on germination requirements of the three species. It is known that a cline in different morphological traits within a genus can be a key factor in the speciation process, e.g. *Coincya* (Gómez-Campo *et al.* 2001). Also, Montesinos-Navarro *et al.* (2012) have observed a clinal variation in seed germination of *Arabidopsis thaliana* related with an altitudinal and climatic gradient from warmer summers and wetter winters to cooler summers and drier winters.

There are differences between *N. longispathus* and *N. alcaracensis* at three levels: physiological (Herranz *et al.* 2013a, b), morphological (Ríos-Ruiz *et al*. 1999) and genetic (Jiménez *et al.* 2009). Therefore, these differences support the taxonomical separation in two different species. On the other hand, many morphological features of *N. yepesii* and the other two *Narcissus* taxa show a large degree of overlap (Table 1), which casts doubt on the taxonomic treatment by Ríos-Ruiz *et al*. (1999). Therefore, with regards to population distribution and morphological appearance, because *N. yepesii* is intermediate to *N. longispathus* and *N. alcaracensis* in many morphological traits, it is of interest to determine which germinative behaviour best describes *N. yepesii* and to relate it with the requirements of the other two close species.

**Table 1.** Morphological traits of each species (Ríos-Ruiz *et al*. 1999; personal observations).

Morphological traits	*N. alcaracensis*	*N. yepesii*	*N. longispathus*
Leaves length (cm)	11–43	19–31	40–60
Floral scape (cm)	10–26	16–35	30–170
Pedicel (mm)	15–46	18–55	40–90
Perianth segments (mm)	14–22	19–25	25–32
Spathe length (mm)	43–60	40–90	60–100
Corona length (mm)	15–23	15–30	14–22
Number of flowers/scape	1–2 (3 rarely)	1	1

In the *Pseudonarcissi* section, all the previous studies report species with some level of MPD (Vandelook and Van Assche 2008; Copete *et al.* 2011a, 2014; Herranz *et al.* 2013a; Newton *et al.* 2015, 2020), meaning freshly matured seeds have underdeveloped dormant embryos (i.e. differentiated but that occupies <50 % of the endosperm length) at the moment of dispersal. A previous laboratory study (unpublished data) showed that embryo length in mature seeds of *N. yepesii* was 1.28 ± 0.05 mm while endosperm length was 3.14 ± 0.05 mm (mean ± standard error [SE], *n* = 25). In addition, these seeds incubated under a wide range of light–temperature conditions (5, 15/4, 20/7, 25/10, 28/14 and 32/18 °C in light/darkness) had not germinated after 30 days. Both results suggest that seeds of *N. yepesii* have an underdeveloped embryo that is physiologically dormant at dispersal time, i.e. this species has some level of MPD. This is also true for the closely related taxa. In *N. alcaracensis* (Herranz *et al.* 2013b), dormancy break and embryo growth just require several months at 5 °C since these seeds have intermediate complex MPD. In *N. longispathus* (Herranz *et al.* 2013a), dormancy break, embryo growth and radicle emergence require warm temperatures followed by cold stratification, corresponding with non-deep complex MPD (Baskin and Baskin 2014).

The present work focuses on verification of the following hypotheses: (i) *N. yepesii* may have different dormancy-breaking requirements than the phylogenetically closely related *Narcissus* species; and (ii) the requirements to break seed dormancy in *Narcissus* populations from the Baetic System may show clinal variation along a northeast–southwest gradient. With a focus on the seed germination of *N. yepesii*, the specific aims of the current study were to investigate: (i) the phenology of embryo growth and radicle and shoot emergence; (ii) dormancy break in seeds buried under natural conditions; (iii) the influence of temperature and light on embryo growth and radicle emergence; (iv) the influence of seed storage duration on embryo growth; (v) the effect of cool temperatures to overcome seed dormancy; (vi) the effect of gibberellic acid (GA_3_ hereafter) on embryo growth; (vii) the influence of cold stratification on shoot emergence; and (viii) induction of dormancy by warm temperatures in non-dormant seeds.

## Materials and Methods

### Seed material

Mature fruits were randomly collected when they changed from green to yellow and began to open for seed dispersal on 7 June 2016 and on 13 June 2017 from a sample around 400 vigorous plants growing in Siles (Jaén province, Spain), 1340 m above sea level (m a.s.l.), 38°23′N, 2°38′W. It is a single homogeneous population compounded by 2000 or 3000 individuals. Ripe capsules were spread out in the laboratory to allow the capsules to open and the seeds to fall out (Newton *et al.* 2013). In recently released seeds, moisture content (fresh weight basis) was 9.4 %. Seeds were dried in the laboratory (22–23 °C) until the initial germination tests began on 1 July 2016 and 2017; at this time seeds were considered to be 0 months old and the seed moisture content was 3.9 %. To analyse seed age effect, seeds were stored dry in paper bags under laboratory conditions: 22–23 °C and 50–60 % air humidity, moisture content remained constant (3.9 %). Unless otherwise stated, experiments were conducted using the 2016 seed lot. Samples of seeds were randomly selected for the several different studies.

### Shadehouse experiments

The purpose of these experiments was to analyse the phenology of dormancy break, i.e. embryo growth, radicle emergence and shoot emergence in *N. yepesii* seeds under near-natural temperature conditions. Therefore, these experiments were conducted in a non-heated metal frame shadehouse (12 threads per cm and 50 % sunlight interception) located at the experimental campus of the Technological School of Agronomy in Albacete (690 m a.s.l., 90 km from Siles). Temperatures were recorded continuously with a TinytagPlus TGP-0020 data-logger (Gemini Data Loggers, Chichester, West Sussex, UK), and mean daily maximum and minimum temperatures were calculated. Pots and trays containing the seeds were filled with a mixture of peat and sand (2:1 v/v) and watered to field capacity once a week throughout the year, with two exceptions. The first exception was to simulate summer water deficit that is common in the Mediterranean area; in July and August, the pots and trays were only watered twice a month. The second exception is that water was withheld when the substratum was frozen in winter. Thus, temperature and soil moisture conditions were similar to those in the natural habitat of *N. yepesii*. Each of the shadehouse experiments was initiated in July and in December to test the effect of warm summer temperatures.

#### Phenology of embryo growth.

On 1 July 2016, eight samples of 30 seeds were mixed with fine-grained sterilized sand, and each sample was placed in a fine-mesh nylon cloth bag. Bags were buried 5-cm deep in an opaque white plastic pot (24 cm of diameter) and placed in the previously described metal-framed shadehouse. Each bag was labelled, but the label was not buried, which made it easy to recover each bag individually. One bag was exhumed each month from August 2016 to March 2017. The bag content was sieved (1-mm mesh size) to separate seeds from sand. Embryos were excised from 25 healthy-looking seeds, i.e. seeds whose embryos were white and firm. More than 95 % of the seeds were viable. Then, embryo length was measured. These values were used in two ways: (i) monthly mean embryo length was calculated to analyse embryo growth throughout the experiment; and (ii) each month these 25 values were grouped into size classes to monitor temporal changes in the distribution of embryo-size structure from August 2016 to March 2017. The study described above was initiated again on 1 December 2016, using four samples of 30 seeds.

Embryos from seeds whose radicles had emerged during burial were recorded as having a critical embryo length (Hidayati *et al.* 2000a; Herranz *et al.* 2010). The critical embryo length for germination is the length of the embryo at the time the seed coat splits but before the radicle emerges (Vandelook and Van Assche 2008). As revealed in preliminary tests in the laboratory, the critical embryo length for germination in *N. yepesii* was 2.75 mm (SE = 0.05 mm, *n* = 40, range = 2.10–3.20 mm) calculated as the mean embryo length in 40 seeds with split seed coat. The E:S ratio is the mean quotient between embryo (E) and endosperm (S) lengths (0.89 ± 0.01). The threshold E:S ratio is a good indicator that the morphological dormancy (MD) component is being broken and seeds are ready to germinate (Copete *et al.* 2011a). It was calculated as the minimum E:S ratio in those 40 seeds (range = 0.76–0.97, where the threshold E:S ratio is 0.76).

#### Phenology of radicle emergence and breaking dormancy phase during burial experiment.

Each sample of 100 seeds was mixed with sand and placed in a nylon permeable bag. In 2016, eight seed samples were buried in a pot (above-mentioned characteristics) on 1 July and four seed samples on 1 December. One bag was exhumed on the first day of each month for 8 and 4 months, respectively. Non-germinated healthy seeds were incubated at 15/4 °C (one of the most favourable temperatures for germination in both *N. longispathus* and *N. alcaracensis*) for 30 days. Seeds recovered from bags were classified into four categories: (i) seeds germinated (radicle emergence) during the burial period; (ii) viable non-dormant seeds that germinated at 15/4 °C; (iii) apparently viable dormant seeds that did not germinate at 15/4 °C and had healthy embryos; and (iv) non-viable seeds that were dead (they were soft and did not contain a firm white embryo).

#### Phenology of shoot emergence.

On 1 July 2016, a sample of 100 seeds of *N. yepesii* was sown 5 mm deep in a tray. Seeds were placed separately from each other to avoid fungal proliferations. Three replicates (three trays with 100 seeds each) were placed in the shadehouse and watered as described above. From July 2016 to March 2019, seed trays were examined once a week, and emergent shoots were counted and removed. The experiment was repeated beginning on 1 December 2016.

### Laboratory experiments

These experiments were carried out in germination chambers (Ibercex model F-4, Madrid, Spain) with a digital temperature and light control system (±0.1 °C, cool white fluorescent light, 25 μmol m^−2^ s^−1^). One hundred seeds (four replicates × 25 seeds) were placed in Petri dishes on two layers of filter paper (Whatman grade 1) moistened with distilled water. Germination tests were conducted under a 12-h daily photoperiod (hereafter ‘light’) and under continuous darkness (hereafter ‘darkness’); darkness was achieved by wrapping Petri dishes in a double layer of aluminium foil (Baskin and Baskin 2014). Dark-incubated seeds were handled under a dim green light (Vandelook *et al.* 2007) to reduce the interruption of the continuous darkness.

Germination tests were conducted using alternating temperature regimes (12/12 h) that simulate mean maximum and mean minimum monthly temperatures during the annual growth cycle at the seed-source region: 15/4 °C, November and March; 20/7 °C, October and April; 25/10 °C, September and May; 28/14 °C, August and June; and 32/18 °C, July. In these 12/12 h alternating temperature regimes, the high temperature coincided with the light phase and the low temperature with darkness to simulate day/night conditions. A constant temperature of 5 °C simulated the mean temperature recorded during winter months: December, January and February. Other low temperatures (9/5 and 10 °C) were chosen because they are within the effective temperature range for cold stratification (0–10 °C) (Nikolaeva 1969). Percentages of germination were based on the number of apparently viable seeds. Embryo growth was calculated throughout each experiment. Embryos excised from 25 seeds at each condition were measured at 30-day intervals.

#### How do temperature and light conditions influence embryo growth?

The purpose of this experiment was to determine which temperatures promote embryo growth; this information is needed to determinate if the MPD level is simple or complex. Twenty 25-seed samples were stratified for 4 months at each of three conditions: (i) cold at 5 °C, (ii) warm at 28/14 °C and (iii) warm followed by cold at 20/7 + 15/4 + 5 + 5 °C (1 month at each temperature). At each condition, ten 25-seed samples were in light and 10 in darkness. Monthly, a 25-seed sample in light and in darkness at each of the three conditions was used to measure mean embryo length (four samples × three treatments). After 4 months of stratification, a 25-seed sample was incubated in light and in darkness at 5, 15/4, 20/7, 25/10, 28/14 and 32/18 °C for 1 month, after which embryos were measured.

#### Does dry storage improve embryo growth?

The aim of this experiment was to verify whether dry storage can promote embryo growth as detected in some species with intermediate complex MPD (Herranz *et al.* 2013b). The effect of seed age on embryo growth was determined in seeds that were dry-stored for 0 (freshly matured seeds) or 20 months. After both dry-storage periods, seeds were incubated in darkness at 5, 15/4 and 20/7 °C and at a 20/7 + 15/4 + 5 + 5 °C sequence of temperatures (1 month for each part of the sequence) for 4 months. At monthly intervals, the embryo length was measured of 25 seeds from each temperature regime.

#### Do low temperatures overcome seed dormancy?

 Seeds dry-stored for 0 or 8 months were cold-stratified at 9/5 and 10 °C for 3–4 months, and after 3 months seeds were incubated in light and darkness at 5, 15/4, 20/7 °C for 1 month. Embryo length was determined monthly and germination percentage was calculated at the end of the experiment.

#### How do temperature–light conditions and seed storage influence radicle emergence of seeds with an almost fully developed embryo?

This experiment was carried out using 0-month-old seeds collected in 2017, and it was repeated when seeds in the same seed sample were 8 and 20 months old. Firstly, two samples of >1200 seeds were stratified for 4 months at 20/7 + 15/4 + 5 + 5 °C (1 month for each part of the temperature regime); one sample was kept in light and the other in darkness. Secondly, from each one of these stratified samples, 12 groups of 100 randomly selected seeds were incubated for 30 days at 5, 15/4, 20/7, 25/10, 28/14 and 32/18 °C in either light or darkness (six temperatures × two light conditions).

#### Does GA_3_ promote embryo growth?

The aim of this experiment was to evaluate the effect of GA_3_ on embryo growth, which is an important trait differentiating levels of MPD since GA_3_ only promotes embryo growth in non-deep or intermediate levels, but not in deep ones (Baskin and Baskin 2014). Samples of 25 seeds were placed in Petri dishes on two sheets of filter paper moistened with a GA_3_ solution (1000 ppm). Then, three dishes each were sealed with Parafilm and placed at each temperature/light condition (5, 25/10 or 28/14 °C in light and in darkness) for 1, 3 or 5 months. The embryo length was measured for one Petri dish from each temperature after 1, 3 and 5 months and to calculate the percentage of seeds whose E:S ratio was higher than the threshold E:S ratio. In a control test, filter paper was moistened with distilled water (GA_3_ 0 ppm), and a dish placed at each of the same six temperature/light conditions; the embryo length was measured only after 5 months.

#### Are seedlings dormant after radicle emergence?

If seedlings are dormant after radicle emergence, the epicotyl would require cold stratification to overcome dormancy (Baskin and Baskin 2014). Two samples of 50 seeds with an emerged radicle (2–3 mm) were placed on two sheets of filter paper moistened with distilled water and incubated in light for 70 days. One of the samples was placed at 20/7 °C and the other sample at 5 °C for 8 weeks and then transferred to 20/7 °C. Shoot emergence was monitored at intervals of 4–5 days.

#### Can warm temperatures induce physiological dormancy after the embryo has grown?

The purpose of this experiment was to determine if warm temperature (28/14 °C) induced dormancy in seeds with an almost fully developed embryo after warm plus cold stratification, and if so, to quantify the period of warm plus cold stratification required for overcoming secondary dormancy. Complete stratification in light lasted 9 months divided into three phases: (i) 4-month dormancy break stratification by warm + cold temperatures (20/7 + 15/4 + 5 + 5 °C); (ii) 1-month dormancy induction by warm temperatures (28/14 °C); and (iii) 4-month secondary-dormancy break stratification by warm + cold temperatures (20/7 + 15/4 + 5 + 5 °C). Germination tests were carried out during stratification to check if seeds were dormant or not dormant after phase 1, after phase 1 + 2, and after phase 1 + 2 + 3 (monthly during phase 3). Each test was conducted with four replicates of 25 seeds at 15/4 °C in both light and darkness for 30 days (optimal conditions for germination in non-dormant seeds). The seed lot was from 2017.

### Statistical analysis

Means and SEs were calculated for percentages of radicle and shoot emergence and for embryo lengths. All the factors analysed were stratification temperature, incubation temperature, light condition during stratification–incubation, time of stratification, concentration of GA_3_ (0 and 1000 ppm) and seed age. An independent statistical analysis was performed in each part of this study. Thus, the effects of the factors involved in each experiment on both embryo length and germination were analysed by multifactor analysis of variance (ANOVA) using Statgraphics centurion XVI. Seed germinability was evaluated by the final cumulative germination percentage of the number of viable seeds. When the effect of a factor was significant, differences were compared by a multiple comparison Tukey test. Prior to analyses, normality (Cochran test) and homoscedasticity (David test) of the data were checked. Values of the final cumulative germination percentages were square-root arcsine transformed.

## Results

### Shadehouse experiments

#### Phenology of embryo growth.

**Figure 2.**
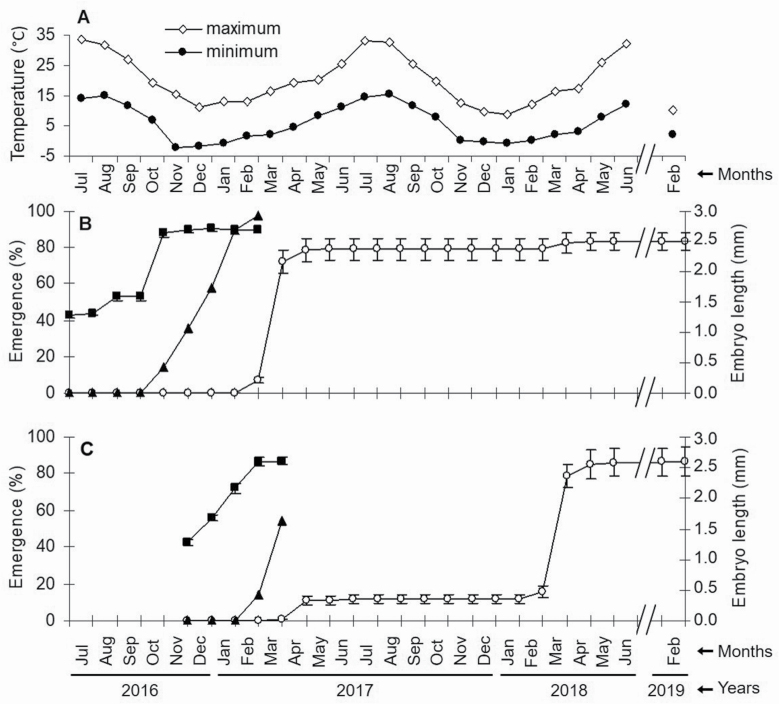
Mean daily minimum and maximum air temperatures (A) and phenology of embryo growth (■) and of root (▲) and seedling shoot (○) emergence from seeds sown on soil in a non-heated shadehouse in July (B) and in December 2016 (C).

For the seeds buried on 1 July 2016 (Fig. 2B), embryos did not grow from 1 July to 1 October; during this period, the mean minimum daily temperature was 14 °C and the mean maximum daily temperature was 31 °C (Fig. 2A). Main part of the embryos (>60 %) was in the three smaller size classes (<1.60 mm) **[see**  **Supporting Information—Fig. S1B****]**. However, embryos grew rapidly completing their development in October (mean minimum temperature 7 °C). Embryo length increased from 1.59 ± 0.07 mm on 1 October to 2.64 ± 0.07 mm on 1 November, reaching the largest size class. Secondly, in seeds buried on 1 December 2016 (Fig. 2C), the embryos grew continuously from December to March; during this period, the mean minimum daily temperature was −0.7 °C and the mean maximum daily temperature was 13 °C. Most of the embryos were not in the >2.20-mm size class until March **[see**  **Supporting Information—Fig. S1C****]**.

#### Phenology of radicle emergence and breaking dormancy phase during burial experiment.

**Figure 3.**
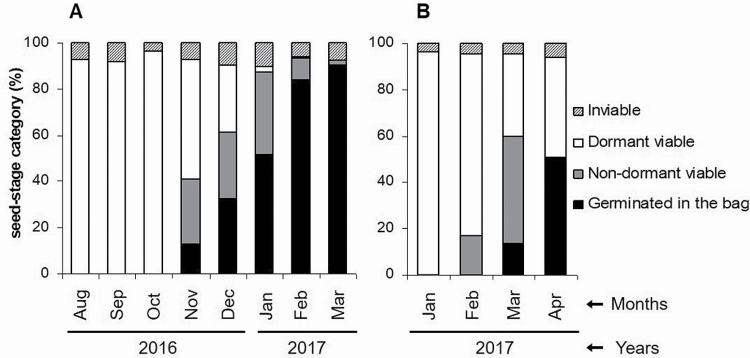
Changes in the percentage of dormant, non-dormant, non-viable and germinated seeds of *N. yepesii* buried on 1 July 2016 (A) and on 1 December 2016 (B) and exhumed monthly for 8 and 4 months, respectively.

 In seeds buried on 1 July 2016 (Fig. 2B), all viable seeds were dormant until October (Fig. 3A), after which radicle emergence began. Cumulative radicle emergence was 13 % in November, 33 % in December, 52 % in January, 84 % in February and 91 % in March. The mean minimum and maximum daily temperatures during this period were −1 and 13 °C, respectively. In seeds buried on 1 December 2016 (Fig. 2C), emergence was 13 % in March and 51 % in April, while 42 % of non-germinated seeds in April were dormant and viable (Fig. 3B).

#### Phenology of shoot emergence.

 In seeds buried on 1 July 2016 (Fig. 2B), the first shoot emergence was observed on 1 March 2017 (7 %); during the previous month the minimum daily temperature was 2 °C and the maximum daily temperature was 16 °C (Fig. 2A). Cumulative shoot emergence was 72 % on 1 April and 79 % on 1 May. A few seedlings (4 %) emerged in the second spring (April 2018). In seeds buried on 1 December 2016 (Fig. 2C), 11 % of shoots had emerged by 1 March 2017 and 79 % emerged in the following spring (April 2018).

### Laboratory experiments

#### How do temperature and light conditions influence embryo growth?

Embryos grew significantly more in darkness than in light at all temperature conditions (*P*-value < 0.05) (Table 2). After 4 months of stratification, the percentages of seeds whose E:S ratio was higher than threshold ratio were 32, 10 and 96 % at 5, 28/14 and the sequence 20/7 + 15/4 + 5 + 5 °C, respectively (Table 2). The highest embryo growth and radicle emergence percentage (74 %) occurred after warm + cold stratification followed by incubation at 15/4 °C in darkness; 21 % of radicles emerged at 20/7 °C in darkness following 4 months at 5 °C. No germination occurred in the warm-stratified seeds subsequently incubated at the six incubation temperatures.

**Table 2.** Effect of (i) cold, (ii) warm and (iii) warm + cold stratifications for 4 months in both light and darkness, followed by incubation for 1 month at: 5, 15/4, 20/7, 25/10, 28/14 or 32/18 °C on embryo growth (mean ± SE, mm, *n* = 25). Values followed by different uppercase letters within a column or different lowercase letters within a row are significantly different at the *P*-value < 0.05 level (Tukey multiple comparisons test). The first number in parentheses is the percentage of radicle emergence, and the second is the percentage of seeds with an E:S ratio greater than the threshold E:S ratio.

	Cold stratification		Warm stratification		Warm + cold stratification	
	5 °C		28/14 °C		20/7 + 15/4 + 5 + 5 °C	
	Light	Darkness	Light	Darkness	Light	Darkness
Stratification duration						
1 month	1.46 ± 0.05^Aab^ (0, 0)	1.36 ± 0.05^Aa^ (0, 0)	1.54 ± 0.03^Aabc^ (0, 0)	1.51 ± 0.05^Aabc^ (0, 0)	1.56 ± 0.05^Abc^ (0, 0)	1.69 ± 0.05^Ac^ (0, 0)
2 months	1.51 ± 0.04^Aa^ (0, 0)	1.58 ± 0.05^ABab^ (0, 0)	1.56 ± 0.04^Aab^ (0, 0)	1.75 ± 0.05^ABbc^ (0, 0)	1.82 ± 0.05^ABc^ (0, 0)	2.20 ± 0.08^Bd^ (0, 32)
3 months	1.61 ± 0.05^Aa^ (0, 0)	1.70 ± 0.07^ABCa^ (0, 4)	1.78 ± 0.07^ABab^ (0, 4)	1.84 ± 0.06^Bab^ (0, 0)	2.05 ± 0.06^BCbc^ (0, 4)	2.29 ± 0.08^Bc^ (0, 46)
4 months	1.64 ± 0.05^Aa^ (0, 0)	1.94 ± 0.10^BCDab^ (0, 32)	1.99 ± 0.05^BCDb^ (0, 0)	1.86 ± 0.08^BCab^ (0, 10)	2.16 ± 0.09^BCDb^ (0, 32)	2.70 ± 0.07^Cc^ (0, 96)
Incubation temperatures						
5 °C	1.73 ± 0.09^Aa^ (0, 10)	2.23 ± 0.09^Dbc^ (8, 44)	2.12 ± 0.06^CDb^ (0, 4)	2.21 ± 0.07^DEb^ (0, 28)	2.54 ± 0.10^Ecd^ (1, 70)	2.75 ± 0.07^Cd^ (44, 96)
15/4 °C	1.73 ± 0.07^Aa^ (6, 8)	2.04 ± 0.08^CDb^ (17, 24)	2.26 ± 0.05^DEbc^ (0, 20)	2.64 ± 0.07^Fd^ (0, 88)	2.34 ± 0.06^CDEc^ (11, 44)	2.71 ± 0.02^Cd^ (74, 100)
20/7 °C	1.72 ± 0.07^Aa^ (4, 4)	2.20 ± 0.09^Db^ (21, 32)	2.41 ± 0.08^Ebc^ (0, 46)	2.37 ± 0.08^EFb^ (0, 40)	2.33 ± 0.07^CDEb^ (1, 40)	2.70 ± 0.05^Cc^ (23, 96)
25/10 °C	1.75 ± 0.07^Aa^ (3, 10)	1.98 ± 0.0^CDab^ (11, 20)	1.96 ± 0.06^BCab^ (0, 8)	2.04 ± 0.06^BCDb^ (0, 20)	2.47 ± 0.09^DEc^ (0, 84)	2.69 ± 0.07^Cc^ (18, 90)
28/14 °C	1.74 ± 0.08^Aa^ (0, 4)	2.08 ± 0.08^Db^ (12, 32)	2.06 ± 0.06^CDb^ (0, 8)	2.18 ± 0.07^DEb^ (0, 40)	2.56 ± 0.11^Ec^ (0, 80)	2.82 ± 0.06^CDc^ (15, 96)
32/18 °C	1.70 ± 0.06^Aa^ (0, 4)	2.23 ± 0.09^Db^ (12, 44)	2.06 ± 0.07^CDb^ (0, 16)	2.15 ± 0.06^CDEb^ (0, 32)	2.53 ± 0.08^Ec^ (0, 76)	3.07 ± 0.06^Dd^ (6, 96)

#### Does dry storage improve embryo growth?

**Figure 4.**
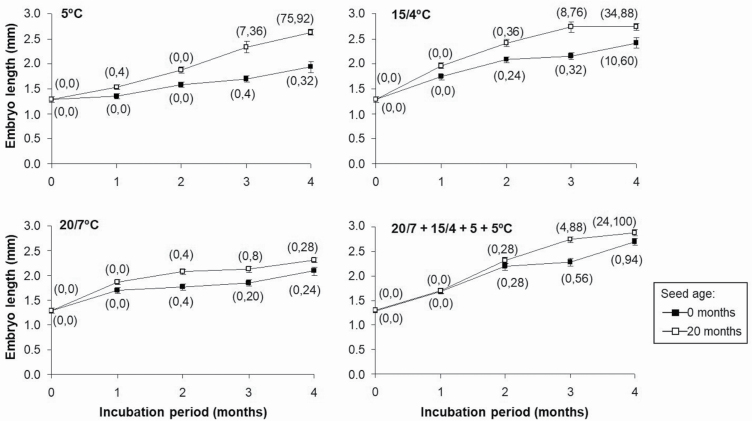
Effect of seed age (0 and 20 months) on embryo growth at different incubation temperatures in darkness for 4 months.

Embryos from freshly matured seeds grew slower than those from 20-month-old seeds (*P*-value < 0.05) (Fig. 4). After 4-month stratification at 5 °C, 32 % of embryos exceeded the threshold E:S ratio in fresh seeds and 92 % in stored seeds; there was no radicle emergence in fresh seeds, and 75 % radicle emergence in the stored seeds. After 4 months at 15/4 °C, 60 % of embryos of fresh seeds (not dry-stored) exceeded the threshold E:S and there was 10 % radicle emergence. After a 3-month stratification at 5 °C, 76 % of embryos of seeds that had been dry-stored for 20 months exceeded the threshold E:S and there was 8 % radicle emergence. At 20/7 °C and at the sequence of temperatures, seed storage only slightly increased the embryo growth.

#### Do cool temperatures overcome seed dormancy?

 At 9/5 and 10 °C, the main embryo growth and germination occurred in 8-month-old seeds incubated in darkness (*P*-value < 0.05) (Table 3). Mean embryo length in 3-month-stratified seeds at 10 °C ranged from 2.04 ± 0.08 mm in 0-month-old seeds in light to 2.51 ± 0.07 mm in 8-month-old seeds in darkness (72 % of radicle emergence) (Table 3). After 4 months of stratification, radicle emergence in 8-month-old seeds was 89 % at 9/5 °C in darkness and 86 % at 10 °C in darkness.

**Table 3.** Embryo growth (mean ± SE, mm, *n* = 25) of seeds cold-stratified for 1–4 months at 9/5 and 10 °C and for seeds given 3 months of cold stratification at both temperatures and then transferred to 5, 15/4 and 20/7 °C for 1 month. Values followed by different uppercase letters within a column or different lowercase letters within a row are significantly different at the *P*-value < 0.05 level (Tukey multiple comparisons test). The first number in parentheses is the percentage of radicle emergence, and the second is the percentage of seeds with an E:S ratio greater than the threshold E:S ratio. Seed age: 0 or 8 months.

	9/5 °C				10 °C			
	0 months		8 months		0 months		8 months	
	Light	Darkness	Light	Darkness	Light	Darkness	Light	Darkness
Stratification duration								
1 month	1.62 ± 0.04^Aa^ (0, 0)	1.79 ± 0.06^Aab^ (0, 0)	1.60 ± 0.04^Aa^ (0, 0)	1.82 ± 0.06^Aab^ (0, 8)	1.64 ± 0.05^Aa^ (0, 0)	1.79 ± 0.05^Aab^ (0, 4)	1.60 ± 0.05^Aa^ (0, 0)	1.91 ± 0.05^Ab^ (0, 0)
2 months	1.92 ± 0.05^Aa^ (0, 12)	2.05 ± 0.08^Aa^ (0, 40)	1.90 ± 0.06^ABa^ (0, 4)	2.37 ± 0.09^Bb^ (0, 44)	1.86 ± 0.07^ABa^ (0, 8)	2.40 ± 0.08^Bb^ (0, 44)	2.04 ± 0.06^Ba^ (0, 16)	2.42 ± 0.08^Bb^ (14, 72)
3 months	2.32 ± 0.09^Bab^ (0, 46)	2.40 ± 0.10^Bb^ (0, 72)	2.21 ± 0.08^BCab^ (0, 40)	2.42 ± 0.06^Bb^ (24, 88)	2.04 ± 0.08^BCa^ (0, 24)	2.42 ± 0.07^Bb^ (0, 76)	2.16 ± 0.11^Bab^ (1, 40)	2.51 ± 0.07^BCb^ (72, 84)
4 months	2.40 ± 0.09^Aabc^ (31, 72)	2.62 ± 0.08^Bc^ (30, 84)	2.28 ± 0.09^Cab^ (14, 40)	2.50 ± 0.07^Babc^ (89, 92)	2.25 ± 0.08^Ca^ (7, 44)	2.46 ± 0.05^BCabc^ (82, 88)	2.26 ± 0.07^BCa^ (8, 48)	2.60 ± 0.05^BCbc^ (86, 100)
Incubation temperatures								
5 °C	2.46 ± 0.09^Bbcd^ (9, 46)	2.68 ± 0.06^Bd^ (57, 88)	2.20 ± 0.09^BCab^ (5, 40)	2.53 ± 0.07^Bcd^ (76, 76)	2.13 ± 0.07^BCa^ (2, 28)	2.62 ± 0.03^BCcd^ (37, 92)	2.34 ± 0.09^BCabc^ (2, 46)	2.59 ± 0.08^BCcd^ (6, 84)
15/4 °C	2.51 ± 0.08^Bab^ (21, 84)	2.70 ± 0.04^Bb^ (72, 96)	2.26 ± 0.10^Ca^ (9, 44)	2.53 ± 0.07^Bab^ (64, 80)	2.29 ± 0.08^Ca^ (2, 44)	2.70 ± 0.06^Cb^ (42, 92)	2.57 ± 0.08^Cab^ (2, 80)	2.58 ± 0.08^BCab^ (36, 84)
20/7 °C	2.48 ± 0.06^Babc^ (8, 76)	2.58 ± 0.08^Bbc^ (38, 76)	2.21 ± 0.08^BCa^ (3, 48)	2.60 ± 0.07^Bbc^ (34, 80)	2.22 ± 0.09^Ca^ (4, 44)	2.56 ± 0.06^BCbc^ (20, 88)	2.34 ± 0.09^BCab^ (2, 46)	2.76 ± 0.05^Cc^ (8, 100)

#### How do temperature*–*light conditions and seed storage influence radicle emergence of seeds with a fully developed embryo?

**Figure 5.**
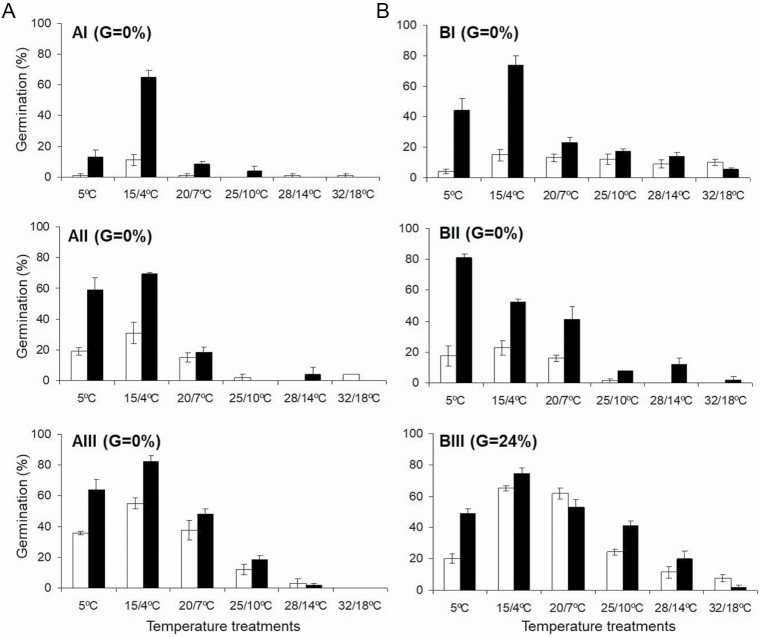
Germination percentages (mean + SE, SE > 2 %) at different incubation temperatures following warm + cold stratification (20/7 + 15/4 + 5 + 5 °C) in light (A) and in darkness (B). Percentage germination (G) during stratification is shown in parentheses. Seeds stored for 0 months (I), 8 months (II) or 20 months (III). White bars show germination percentages during incubation in light conditions and black bars show germination percentages during incubation in darkness conditions.

 Radicle emergence increased significantly in 20-month-old seed stratified and incubated in darkness. The optimal incubation temperature was 15/4 °C (*P*-value < 0.001) with germination ranging from 52 to 82 % (Fig. 5). The lowest germination percentages (<20 %) were observed at 28/14 and 32/18 °C.

#### Does GA_3_ improve embryo growth?

There were no significant differences (*P*-value > 0.05) between seeds tested with GA_3_ (1000 ppm) and distilled water (control) at 25/10 and 28/14 °C (Table 4). After 5 months of stratification at 5 °C in darkness, embryo length was 2.35 ± 0.11 mm with GA_3_ and 2.23 ± 0.09 mm in the control (the difference was not significant); radicle emergence was 27 % with GA_3_ and 8 % in the control.

**Table 4.** Effect of GA_3_ (1000 ppm) on embryo growth (mean ± SE, mm, *n* = 25). Values followed by different uppercase letters within a column or different lowercase letters within a row are significantly different at the *P*-value < 0.05 level (Tukey multiple comparisons test). The first number in parentheses is the percentage of radicle emergence, and the second is the percentage of seeds with an E:S ratio greater than the threshold E:S ratio.

	5 °C		25/10 °C		28/14 °C	
Incubation (months)	Light	Darkness	Light	Darkness	Light	Darkness
1	1.40 ± 0.05^Aa^ (0, 0)	1.51 ± 0.06^Aab^ (0, 0)	1.60 ± 0.06^Aab^ (0, 0)	1.65 ± 0.05^Ab^ (0, 0)	1.66 ± 0.07^Ab^ (0, 0)	1.62 ± 0.07^Aab^ (0, 0)
3	1.65 ± 0.07^ABa^ (0, 10)	2.06 ± 0.12^BCb^ (0, 32)	1.76 ± 0.06^ABab^ (0, 0)	1.87 ± 0.07^ABab^ (0, 4)	1.84 ± 0.06^Aab^ (0, 10)	1.96 ± 0.06^Bb^ (0, 0)
5	1.75 ± 0.07^Ba^ (4, 10)	2.35 ± 0.11^Cc^ (27, 46)	1.87 ± 0.05^Bab^ (0, 0)	2.07 ± 0.06^Bbc^ (0, 10)	2.16 ± 0.07^Bbc^ (0, 24)	1.96 ± 0.06^Bab^ (0, 4)
Control (0 ppm)	1.73 ± 0.09^Ba^ (0, 10)	2.23 ± 0.09^Cb^ (8, 44)	1.98 ± 0.07^Bab^ (0, 0)	2.05 ± 0.07^Bab^ (0, 10)	2.18 ± 0.08^Bb^ (0, 24)	2.11 ± 0.08^Bb^ (0, 20)

#### Are seedlings dormant after radicle emergence?

**Figure 6.**
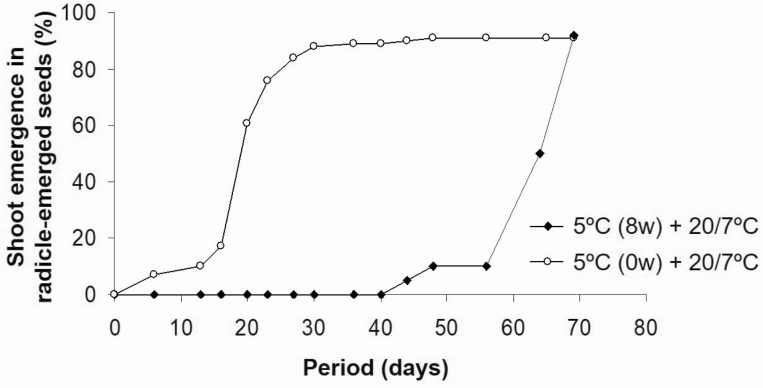
Shoot growth in germinated seeds of *N. yepesii* at 20/7 °C (10 weeks) or at 5 °C (8 weeks) followed by 20/7 °C (2 weeks).

An 80 % of shoot emergence was reached in radicle-emerged seeds after 67 or 24 days depending on whether seeds were previously cold-stratified or not (Fig. 6). Shoots began to emerge after 6 days at 20/7 °C or after 44 days at 5 °C; however, after this delay, emergence rate was similar in both experiments, around 90 % of shoots emerged within 30 days.

#### Can warm temperatures induce physiological dormancy after the embryo has grown?

Seed receiving warm + cold stratification and then incubated in light at 15/4 °C had 55 % radicle emergence, and seeds receiving similar stratification treatment but incubated in darkness had 75 % radicle emergence (Table 5). There was no germination of seed receiving warm + cold + warm stratification and then incubated in light at 15/4 °C. Seeds receiving similar stratification and temperature treatment, but incubated in darkness had 20 % germination. Seeds receiving (warm + cold) + warm + (warm + cold) and transferred monthly during the last 4-month stratification to incubation conditions had low radicle emergence until the last of the 4 months when warm + cold sequence was completed; germination reached 73 % at 15/4 °C in light.

**Table 5.** Induction of secondary dormancy by warm stratification (28/14 °C). Percentages of radicle emergence (mean ± SE, *n* = 4) after incubation at 15/4 °C in light and darkness following different stratification sequences. Seed age = 20 months. (W + C) = 20/7 + 15/4 + 5 + 5 °C, 30 days at each temperature. W = 28/14 °C (30 days). ---------^a^ = no data.

Stratification	Incubation at 15/4 °C	
Temperature sequences	Light	Darkness
(W + C)	55 ± 3	75 ± 3
(W + C) + W	0 ± 0	20 ± 3
(W + C) + W + (20/7 °C)	0 ± 0	---------^a^
(W + C) + W + (20/7 + 15/4 °C)	0 ± 0	---------^a^
(W + C) + W + (20/7 + 15/4 + 5 °C)	4 ± 0	---------^a^
(W + C) + W + (20/7 + 15/4 + 5 + 5 °C)	73 ± 2	---------^a^

## Discussion

Diverse evolutionary processes with different selection pressures have produced a wide array of ecophysiological traits, such as different classes of seed dormancy (Walck and Hidayati 2004; Baskin and Baskin 2014). Within the genus *Narcissus,* there are species with fully developed embryos at dispersal time (*Jonquillae* and *Tazettae* sections) and species with underdeveloped embryos at dispersal time (*Bulbocodii* and *Pseudonarcissi* sections) (Santiago 2012). According to Forbis *et al.* (2002), early diverging species have an underdeveloped embryo, and therefore exhibit MD or MPD, while the other dormancy classes are predicted to be derived from them (Baskin and Baskin 2004; Willis *et al.* 2014). Therefore, the *Pseudonarcisii* section occupies an ancestral place in the trait evolution process of the genus.

**Figure 7.**
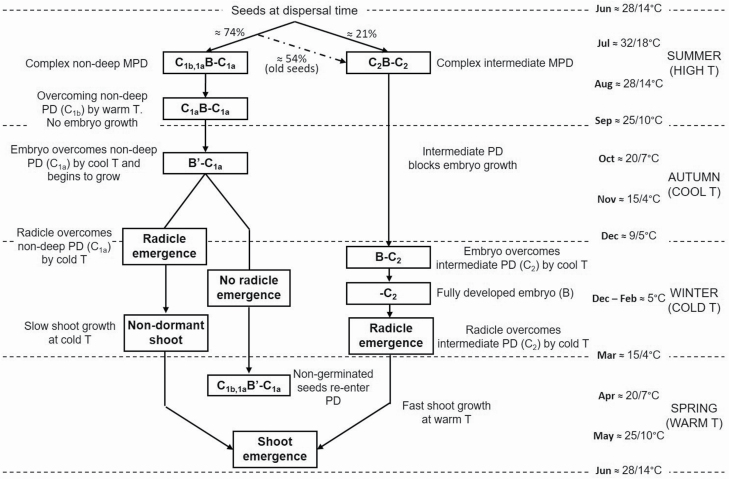
Conceptual model of dormancy break in seeds of *N. yepesii*. Temperature (T), underdeveloped embryo (B), PD (C), non-deep PD (subscript 1), intermediate PD (subscript 2), warm stratification requirement (subscript b), cold stratification requirement (subscript a). Some seeds re-enter MPD, where B′ indicates embryos have grown, but have not fully developed.

*Narcissus yepesii*, belonging to this last section, has an underdeveloped embryo, whose length is 1.28 ± 0.05 mm in mature seeds, which is 40 % of seed length. The seed is not able to germinate unless the embryo reaches at least the threshold length (2.1 mm), equivalent to 75 % of seed length. The lapse of time required for embryo growth and radicle emergence in optimal conditions is much longer than 30 days since a stratification period is necessary. This fact indicates that the embryo has to overcome both components of MPD: (i) MD and (ii) physiological dormancy (PD) (Baskin and Baskin 2014). Figure 7 summarizes the complex dormancy in *N. yepesii*.

The embryo growth in *N. yepesii* seeds occurs mainly during cold winter temperatures (5 °C) following warm summer/autumn temperatures (Table 2) or at cool temperatures (9/5 and 10 °C) (Table 3). Therefore, the level of MPD is complex since embryo growth occurs from 0 to 10 °C (Nikolaeva 1969; Baskin and Baskin 2014). The most favourable temperature sequence is a warm stratification (20/7 + 15/4 °C, 1 month each) followed by 2 months at 5 °C, whereupon 96 % of seeds have an E:S ratio ≥ threshold E:S ratio, (Table 2), meaning that the morphological component of MPD has been overcome (Copete *et al.* 2011a). After the embryo grew, 74 % of the seeds incubated in darkness to autumn temperatures (15/4 °C) germinated.

To gain a better understanding of physiological component of MPD in *N. yepesii*, the effects of GA_3_ and of seed storage on overcoming dormancy were analysed. Gibberellic acid produced some stimulation of germination at low temperatures (5 °C in darkness) (Table 4), and dry laboratory storage had a significant positive influence on radicle emergence in 20-month-old seeds following a warm + cold stratification (Fig. 5). These results strongly support non-deep complex MPD in most of the seeds, which also occurs in seeds of *N. longispathus* (Herranz *et al.* 2013a).

The embryo growth was reduced when there was not a previous warm period before cold stratification. In seeds stratified for 4 months at 9/5 or 10 °C, 84 and 88 % of embryos reached the threshold E:S ratio, respectively (Table 3). The explanation for high percentages is that both 10 and 9 °C (12 h day^−1^) are within the range of temperatures effective to overcome dormancy in some species whose requirement is warm followed by cold or moderately warm. A similar response has been found in *Gagea lutea* (Kondo *et al.* 2004) with deep simple epicotyl MPD, and also in *Symphoricarpos orbiculatus* (Flemion and Parker 1942) with non-deep complex MPD. Seeds of both of these species exhibit embryo growth and radicle emergence at 10 °C.

The optimal temperature for many species that require cold stratification to break PD is 5 °C (Stokes 1965; Baskin and Baskin 2014), but only 32 % of embryos of *N. yepesii* reached the threshold E:S ratio after 4-month stratification at 5 °C (Table 2), and only 21 % of these stratified seeds germinated when incubated at 20/7 °C in darkness. This portion of the seeds did not require a previous warm stratification; thus, they have deep or intermediate complex MPD (Baskin and Baskin 2014), whose physiological component is overcome with a long cold period (Nikolaeva 1969). A deep level of complex MPD could be suggested by the null effect of GA_3_ on embryo growth after 5 months of incubation at 25/10 °C or 28/14 °C (Table 4) (Walck and Hidayati 2004; Vandelook *et al.* 2007). However, seeds with fully developed embryos and intermediate PD may, or may not respond to GA_3_ (Baskin and Baskin 2004). Attending to dry storage, this shortened the cold stratification period required for dormancy break (Fig. 4), which indicates the presence of intermediate PD. Radicle emergence was 75 % in seeds of *N. yepesii* stored dry for 20 months and then incubated at 5 °C for 4 months without a previous warm stratification, whereas 0-month seeds incubated under the same conditions exhibited slow embryo growth and no germination (Fig. 4). Therefore, 21 % of the 0-month seeds had intermediate complex MPD, which is like seeds of the closely related *N. alcaracensis* (Herranz *et al.* 2013b).

The unusual existence of several complex MPD levels within a single seed population has been shown in *Sanicula canadensis* and *S. gregaria* (Hawkins *et al*. 2010). Seeds with several levels of MPD have been documented in *Lonicera etrusca* (Santiago *et al.* 2013) and in *L. maackii* (Hidayati *et al.* 2000a); in each study, the sum of seed percentages with different breaking dormancy requirements was >100 %. This overlap is because there are some seeds with different levels of MPD. In *N. yepesii*, ~74 % of the seeds have non-deep complex MPD, and 21 % have intermediate complex MPD. However, the percentage with intermediate complex MPD (require only cold stratification for dormancy break) increased to 75 % after 20 months of dry storage. Thus, ~54 % (75 − 21 %) of the seeds could have both levels of MPD since they can germinate with: (i) warm + cold (non-deep complex), or (ii) cold + storage (intermediate complex). Therefore, the seed fraction having complex non-deep MPD at dispersal time (i.e. warm + cold stratification requirements), but without enough soil moisture during summer/autumn to be warm-stratified, would be able to germinate at the end of the next cold wet winter after long exposure to only low stratification temperatures. This is because of the positive effect of seed dry storage during summer/autumn drought on germination.

In relation to shadehouse experiments, values of germination close to 100 % have been reached under natural conditions in contrast to laboratory tests. The coexistence of both dormancy levels implies a high final germination but over a period of several months, from November to March in seeds sown in July (Fig. 2B). Firstly, non-deep dormant seeds germinate from November to January after a warm summer followed by a cool autumn, and then cold winter temperatures. Secondly, those seeds with an intermediate dormancy germinate from February to March because a longer cold period is required to overcome dormancy. Consequently, requirements of both levels are satisfied under natural conditions, and so final germination is close to 100 %. However, when seeds are sown in December after having been stored for 5 months, germination is only 54 % (Fig. 2C), since these seeds have not received warm stratification; however, there is a positive seed storage effect.

The epicotyl is non-dormant in germinated seeds of *N. yepesii* since 80 % of shoots emerge from emerged-radicle seeds throughout 25 days of incubation at spring temperatures (20/7 °C) without a previous cold stratification (Fig. 6). The time (2 months) between radicle and shoot emergence could be due to low winter temperatures in the natural habitat, which means that a rapid shoot emergence can be avoided; this is similar to other species with non-deep complex MPD such as *Merendera montana* (Copete *et al.* 2011b).

Radicle emergence in *N. yepesii* seeds is promoted by darkness rather than light conditions (Fig. 5). Although this prevents the formation of a soil seed bank, the non-germinated seeds during the first spring after seed dispersal can re-enter secondary dormancy through the effect of warm summer temperatures (Table 5); this occurs in *N. longispathus* and *N. alcaracensis* (Herranz *et al.* 2016), and the seeds of these species will only be able to germinate in the second spring (Figs 2 and 7).

In view of the obtained results, the proposed hypothesis that *N. yepesii* may have different dormancy-breaking requirements to the phylogenetically closely related *Narcissus* species is not supported. In fact, remarkably the level of MPD present in seeds of *N. alcaracensis* and that in *N. longispathus* are both present in seeds of *N. yepesii*. However, the second hypothesis is satisfied since we have verified that seed dormancy breaking requirements in *Narcissus* populations from the Baetic System mountains show clinal variation in a northeast–southwest gradient from intermediate to non-deep complex, through the unusual coexistence of both levels in *N. yepesii* plants living in an intermediate location of this geographical range (Fig. 1). Such a co-occurrence of dormancy levels should reflect the adaptation to the particular environmental conditions (Donohue *et al.* 2010).

Based on chromosomic studies, Fernandes (1975) showed *N. nevadensis* as being the most early diverging species from the *Pseudonarcissi* section. Afterwards, Herranz *et al.* (2013a) assigned intermediate complex MPD to the seeds of this species and suggested this dormancy level (3–4 months of cold stratification) as a possible way leading to non-deep complex MPD (2 months of warm + 2 months of cold stratification) throughout the adaptation to wetter environments, i.e. with an increase of annual precipitation from 750 to 980 mm, and to warmer winters, i.e. with an increase of mean temperature of the coldest month from 3.5 to 5.5 °C in the south-west area (Rivas-Martínez 1987). The anthropogenic climate change, with a progressive reduction of the cold period in the natural environment (Parmesan and Hanley 2015), could accentuate this tendency. However, the confirmation of this evolutionary scheme between levels of MPD requires delving into the knowledge of the phylogeny in the *Narcissus* group. Although Medrano *et al.* (2014) have analysed the genetic structure of daffodil populations in the Baetic System, further studies are still needed to evaluate the taxonomic status of some of these populations.

A similar model occurs in species of *Osmorhiza* (Apiaceae) from North America. So, *O. occidentalis* and *O. depauperata* in the West have deep complex MPD (ancestral dormancy condition), whereas *O. claytonii* and *O. longistylis* in the East have non-deep complex MPD (derived condition) (Walck and Hidayati 2004). Nevertheless, a species with both levels of MPD, which could play the role of *N. yepesii* in this study, was not identified in that model.

A dormancy cline related to altitude (m a.s.l.), mean annual temperature (°C) and summer precipitation (mm) has been shown in *Centaurium somedanum* (Gentianaceae), an endemic perennial herb of north-western Spain whose seeds have non-deep simple MPD (Fernández-Pascual *et al.* 2013). Nevertheless, this is the first study to show a gradient between levels of intermediate complex and non-deep complex MPD. Thus, the seed dormancy evolution across the cline from one level of MPD to another level of MPD, as adaptation to environmental changes, can be a key factor determining the course of the plant trait evolution process in species with underdeveloped embryos.

## Supporting Information

The following additional information is available in the online version of this article—

**Figure S1.** Shows changes in size class distribution of embryos in seeds of *Narcissus yepesii*: at seed dispersal time (A), sown in July (B) and in December (C) and recovered each month for 4 months.

## Supplementary Material

plaa060_suppl_Supplementary_Materials

plaa060_suppl_Supplementary_Figure_1

## Data Availability

All data are available in the online version of the article as **Supporting Information**.

## Notes

**Plants, Ecosystems & Climate. Chief Editor:** Mary Heskel

## References

[CIT0001] Baskin CC, Baskin JM 2014 Seeds: ecology, biogeography, and evolution of dormancy and germination, 2nd edn. San Diego, CA: Academic/Elsevier.

[CIT0002] Baskin JM, Baskin CC 2004 A classification system for seed dormancy. Seed Science Research 14:1–16.

[CIT0003] Blanchard J 1990 Narcissus: a guide to wild daffodils. Lye End Link, Woking, UK: AGS.

[CIT0004] Copete E, Herranz JM, Copete MA, Baskin JM, Baskin CC 2011b Non-deep complex morphophysiological dormancy in seeds of the Iberian Peninsula endemic geophyte *Merendera montana* (Colchicaceae). Seed Science Research 21:267–281.

[CIT0005] Copete E, Herranz JM, Copete MA, Ferrandis P 2014 Interpopulation variability on embryo growth, seed dormancy break, and germination in the endangered Iberian daffodil *Narcissus eugeniae* (Amaryllidaceae). Plant Species Biology 29:e72–e84.

[CIT0006] Copete E, Herranz JM, Ferrandis P, Baskin CC, Baskin JM 2011a Physiology, morphology and phenology of seed dormancy break and germination in the endemic Iberian species *Narcissus hispanicus* (Amaryllidaceae). Annals of Botany 107:1003–1016. 21335326 10.1093/aob/mcr030 PMC3080619

[CIT0007] Donohue K, Rubio de Casas R, Burghardt L, Kovach K, Willis CG 2010 Germination, postgermination, adaptation, and species ecological ranges. The Annual Review of Ecology, Evolution and Systematics 41:293–319.

[CIT0008] Fernandes A 1975 L’evolution chez le genre *Narcissus* L. Anales Instituto Botanico Cavanilles 32:843–872.

[CIT0009] Fernández-Pascual E, Jiménez-Alfaro B, Caujapé-Castells J, Jaén-Molina R, Díaz TE 2013 A local dormancy cline is related to the seed maturation environment, population genetic composition and climate. Annals of Botany 112:973–945. 23864001 10.1093/aob/mct154 PMC3747807

[CIT0010] Flemion F, Parker A 1942 Germination studies of seeds of *Symphoricarpos orbiculatus*. Contributions from the Boyce Thompson Institute 12:301–307.

[CIT0011] Forbis TA, Floyd SK, Queiroz A 2002 The evolution of embryo size in angiosperms and other seed plants: implications for the evolution of seed dormancy. Evolution 56:2112–2125. 12487343 10.1111/j.0014-3820.2002.tb00137.x

[CIT0012] Gómez-Campo C, Herranz-Sanz JM, Montero-Riquelme F 2001 The genus *Coincya* Rouy (Cruciferae) in south-central Spain revisited: a morphometric analysis of population structure. Botanical Journal of the Linnean Society 135:125–135.

[CIT0013] Grubb PJ 1977 The maintenance of species-richness in plant communities: the importance of the regeneration niche. Biological Reviews 52:107–145.

[CIT0500] Hawkins TS, Baskin CC, Baskin JM 2010 Morphophysiological dormancy in seeds of three eastern North American Sanicula species (Apiaceae subf. Saniculoideae): evolutionary implications for dormancy break. Plant Species Biology 25:103–113.

[CIT0014] Hernández-Bermejo E, Prados J, Benavente A, Díaz A, Herrera-Molina F, Garrido A, Luque P 2006 *Narcissus longispathus*. The IUCN Red List of Threatened Species 2006: e.T61602A12519309.

[CIT0015] Herranz JM, Copete E, Copete MA, Márquez J, Ferrandis P 2016 Dormancy induction by summer temperatures and/or desiccation in imbibed seeds of trumpet daffodils *Narcissus alcaracensis* and *N. longispathus* (Amaryllidaceae). Plant Biology 19:46–52. 27094365 10.1111/plb.12467

[CIT0016] Herranz JM, Copete E, Ferrandis P 2013a Nondeep complex morphophysiological dormancy in *Narcissus longispathus* (Amaryllidaceae): implications for evolution of dormancy levels within section *Pseudonarcissi*. Seed Science Research 23:141–155.

[CIT0017] Herranz JM, Copete MA, Ferrandis P 2013b Environmental regulation of embryo growth, dormancy break and germination in *Narcissus alcaracensis* (Amaryllidaceae), a threatened endemic Iberian daffodil. American Midland Naturalist 169:147–167.

[CIT0018] Herranz JM, Ferrandis P, Martínez-Duro E 2010 Seed germination ecology of the threatened endemic Iberian *Delphinium fissum* subsp. *sordidum* (Ranunculaceae). Plant Ecology 211:89–106.

[CIT0019] Hidayati SN, Baskin JM, Baskin CC 2000a Dormancy-breaking and germination requirements of seeds of four *Lonicera* species (Caprifoliaceae) with underdeveloped spatulate embryos. Seed Science Research 10:459–469.

[CIT0020] Hidayati SN, Baskin JM, Baskin CC 2000b Morphophysiological dormancy in seeds of two North American and one Eurasian species of *Sambucus* (Caprifoliaceae) with underdeveloped spatulate embryos. American Journal of Botany 87:1669–1678. 11080118

[CIT0021] Jiménez JF, Sánchez-Gómez P, Guerra J, Molins A, Rosselló JA 2009 Regional speciation or taxonomic inflation? The status of several narrowly distributed and endangered species of *Narcissus* using ISSR and nuclear ribosomal ITS markers. Folia Geobotanica 44:145–158.

[CIT0022] Juan-Vicedo J, Ríos-Ruiz S, García-Murillo PG 2018 *Narcissus alcaracensis*. The IUCN Red List of Threatened Species 2018: e.T61600A57488304.

[CIT0023] Kondo T, Miura T, Okubo N, Shimada M, Baskin C, Baskin J 2004 Ecophysiology of deep simple epicotyl morphophysiological dormancy in seeds of *Gagea lutea* (Liliaceae). Seed Science Research 14:371–378.

[CIT0024] Medrano M, López-Perea E, Herrera CM 2014 Population genetics methods applied to a species delimitation problem: endemic trumpet daffodils (*Narcissus* section *Pseudonarcissi*) from the southern Iberian Peninsula. International Journal Plant Science 175:501–517.

[CIT0025] Montesinos-Navarro A, Picó FX, Tonsor SJ 2012 Clinal variation in seed traits influencing life cycle timing in *Arabidopsis thaliana*. Evolution 66:3417–3431. 23106707 10.1111/j.1558-5646.2012.01689.x

[CIT0026] Newton RJ, Hay FR, Ellis RH 2013 Seed development and maturation in early spring-flowering *Galanthus nivalis* and *Narcissus pseudonarcissus* continues post-shedding with little evidence of maturation in planta. Annals of Botany 111:945–955. 23478943 10.1093/aob/mct051 PMC3631335

[CIT0027] Newton RJ, Hay FR, Ellis RH 2015 Ecophysiology of seed dormancy and the control of germination in early spring-flowering *Galanthus nivalis* and *Narcissus pseudonarcissus* (Amaryllidaceae). Botanical Journal of the Linnean Society 177:246–262.

[CIT0028] Newton RJ, Hay FR, Ellis RH 2020 Temporal patterns of seed germination in early spring-flowering temperate woodland geophytes are modified by warming. Annals of Botany. doi:10.1093/aob/mcaa025. PMC7262466 32055829

[CIT0029] Nikolaeva MG 1969 Physiology of deep dormancy in seeds. Leningrad, Russia: Izdatel’stvo ‘Nauka’ (Translated from Russian by Z. Shapiro, National Science Foundation, Washington, DC.).

[CIT0030] Parmesan C, Hanley ME 2015 Plants and climate change: complexities and surprises. Annals of Botany 116:849–864. 26555281 10.1093/aob/mcv169 PMC4640131

[CIT0031] Ríos-Ruiz S, Rivera D, Alcaraz F, Obón de Castro C 1999 Three new species of *Narcissus* L. subgenus Ajax Spach (Amaryllidaceae), restricted to the meadows and forests of south-eastern Spain. Botanical Journal of the Linnean Society 131:153–165.

[CIT0032] Rivas-Martínez S 1987 Memoria del mapa de series de vegetación de España. ICONA, Serie Técnica. Madrid: Ministerio Agricultura, Pesca y Alimentación.

[CIT0033] Santiago A 2012 Ecología germinativa de once especies de flora singular. PhD Thesis, University of Castilla-La Mancha, Spain.

[CIT0034] Santiago A, Herranz JM, Copete E, Ferrandis P 2013 Species-specific environmental requirements to break seed dormancy: implications for selection of regeneration niches in three *Lonicera* (Caprifoliaceae) species. Botany 91:1–9.

[CIT0501] Stokes P 1965 Temperature and seed dormancy. In: Ruhland W, ed. Encyclopaedia of plant physiology, Vol. 15, part 2. New York: Springer, 746–803.

[CIT0035] Vandelook F, Bolle N, Van Assche JA 2007 Seed dormancy and germination of the European *Chaerophyllum temulum* (Apiaceae), a member of a trans-Atlantic genus. Annals of Botany 100:233–239. 17556382 10.1093/aob/mcm090 PMC2735313

[CIT0036] Vandelook F, Van Assche JA 2008 Temperature requirements for seed germination and seedling development determine timing of seedling emergence of three monocotyledonous temperate forest spring geophytes. Annals of Botany 102:865–875. 18757880 10.1093/aob/mcn165 PMC2712388

[CIT0037] Walck JL, Hidayati SN 2004 Germination ecophysiology of the western North American species *Osmorhiza depauperata* (Apiaceae): implications of preadaptation and phylogenetic niche conservatism in seed dormancy evolution. Seed Science Research 14:387–394.

[CIT0038] Walck JL, Hidayati SN, Okagami N 2002 Seed germination ecophysiology of the Asian species *Osmorhiza aristata* (Apiaceae): comparison with its North American congeners and implications for evolution of types of dormancy. American Journal of Botany 89:829–835. 21665683 10.3732/ajb.89.5.829

[CIT0039] Willis CG, Baskin CC, Baskin JM, Auld JR, Venable DL, Cavender-Bares J, Donohue K, Rubio de Casas R; The NESCent Germination Working Group 2014 The evolution of seed dormancy: environmental cues, evolutionary hubs, and diversification of the seed plants. The New Phytologist 203:300–309. 24684268 10.1111/nph.12782

[CIT0040] Zonneveld BJM 2008 The systematic value of nuclear DNA content for all species of *Narcissus* L. (Amaryllidaceae). Plant Systematics and Evolution 275:109–132.

