# Supplementary figures and images for: Seed germination in Narcissus yepesii (Amaryllidaceae): clinal variation in the morphophysiological dormancy levels
Source: AoB Plants. 2020 Nov 17;12(6):plaa060. doi: 10.1093/aobpla/plaa060 (PMC7774471; doi:10.1093/aobpla/plaa060)

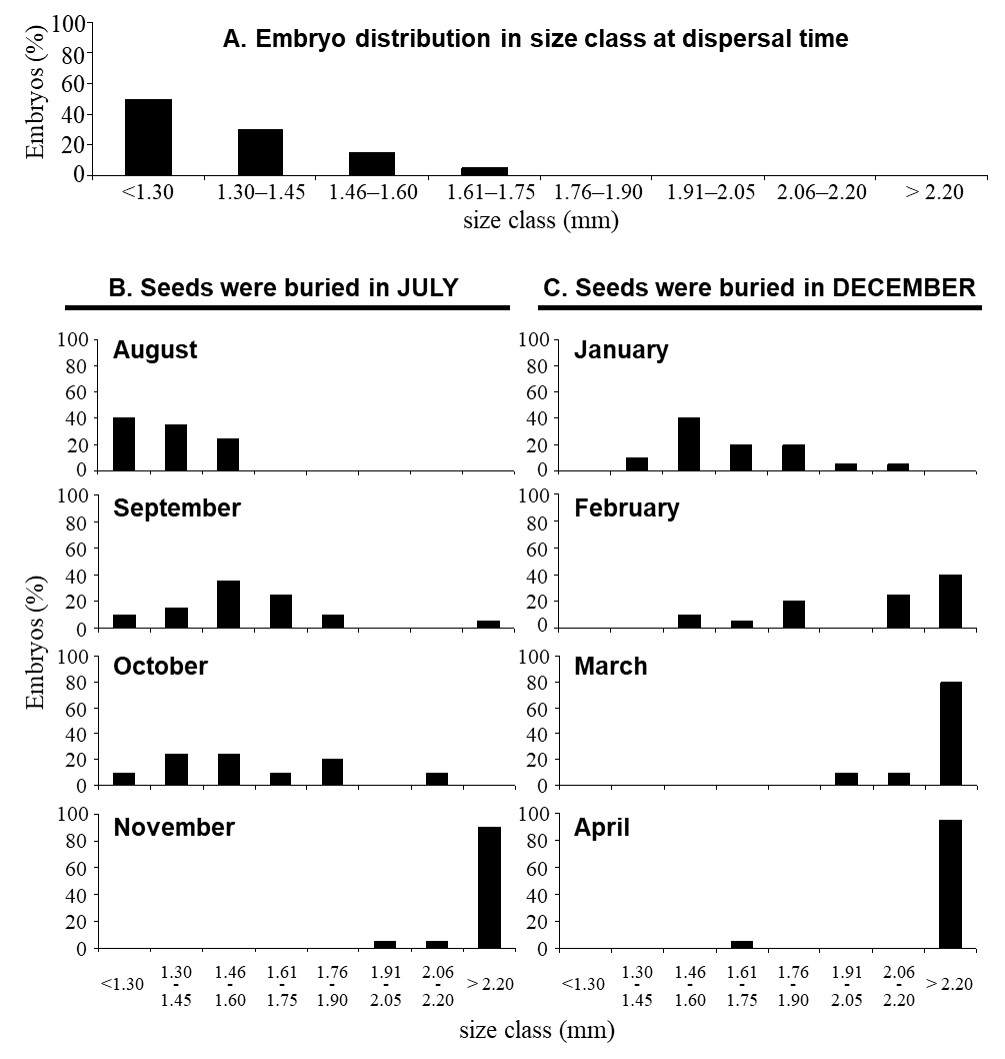

Supplement: plaa060_suppl_Supplementary_Figure_1 [file plaa060_suppl_supplementary_figure_1.jpeg]
